# A discrete cluster of urinary biomarkers discriminates between active systemic lupus erythematosus patients with and without glomerulonephritis

**DOI:** 10.1186/s13075-016-1120-0

**Published:** 2016-10-04

**Authors:** Carolina Landolt-Marticorena, Stephenie D. Prokopec, Stacey Morrison, Babak Noamani, Dennisse Bonilla, Heather Reich, James Scholey, Carmen Avila-Casado, Paul R. Fortin, Paul C. Boutros, Joan Wither

**Affiliations:** 1 Krembil Research Institute, University Health Network, Toronto, Canada; 2 Informatics and Bio-computing Program, Ontario Institute for Cancer Research, Toronto, Ontario Canada; 3 Department of Nephrology, University Health Network, University of Toronto Faculty of Medicine, Toronto, Canada; 4 Department of Pathology, University of Toronto, Toronto General Hospital, University Health Network, Toronto, Canada; 5 Centre de recherche du CHU de Québec – Université Laval and Department of Medicine, CHU de Québec – Université Laval, Quebec City, Canada; 6 Department of Pharmacology & Toxicology, University of Toronto, Toronto, Ontario Canada; 7 Department of Medical Biophysics, University of Toronto, Toronto, Ontario Canada; 8 Division of Rheumatology, University Health Network, Toronto, Canada; 9 Departments of Medicine and Immunology, University of Toronto, Toronto, Canada; 10 Toronto Western Hospital, 1E-420, 399 Bathurst Street, Toronto, ON M5T 2S8 Canada

**Keywords:** Systemic lupus erythematosus, Glomerulonephritis, Urinary biomarkers, Renal biopsy

## Abstract

**Background:**

Management of lupus nephritis (LN) would be greatly aided by the discovery of biomarkers that accurately reflect changes in disease activity. Here, we used a proteomics approach to identify potential urinary biomarkers associated with LN.

**Methods:**

Urine was obtained from 60 LN patients with paired renal biopsies, 25 active non-LN SLE patients, and 24 healthy controls. Using Luminex, 128 analytes were quantified and normalized to urinary creatinine levels. Data were analyzed by linear modeling and non-parametric statistics, with corrections for multiple comparisons. A second cohort of 33 active LN, 16 active non-LN, and 30 remission LN SLE patients was used to validate the results.

**Results:**

Forty-four analytes were identified that were significantly increased in active LN as compared to active non-LN. This included a number of unique proteins (e.g., TIMP-1, PAI-1, PF4, vWF, and IL-15) as well as known candidate LN biomarkers (e.g., adiponectin, sVCAM-1, and IL-6), that differed markedly (>4-fold) between active LN and non-LN, all of which were confirmed in the validation cohort and normalized in remission LN patients. These proteins demonstrated an enhanced ability to discriminate between active LN and non-LN patients over several previously reported biomarkers. Ten proteins were found to significantly correlate with the activity score on renal biopsy, eight of which strongly discriminated between active proliferative and non-proliferative/chronic renal lesions.

**Conclusions:**

A number of promising urinary biomarkers that correlate with the presence of active renal disease and/or renal biopsy changes were identified and appear to outperform many of the existing proposed biomarkers.

**Electronic supplementary material:**

The online version of this article (doi:10.1186/s13075-016-1120-0) contains supplementary material, which is available to authorized users.

## Background

Nephritis and its treatment constitute one of the major causes of morbidity and mortality in systemic lupus erythematosus (SLE). Approximately 50–60 % of SLE patients will develop lupus nephritis (LN), with 30 % of these patients developing significant renal impairment, culminating in end-stage renal disease in 15 % of patients [1, 2]. The clinical course of LN is highly variable, being marked by unpredictable flares and variable responses to treatment [3, 4]. As treatment of LN results in significant immediate (e.g., infection) and delayed (e.g., avascular necrosis and cardiovascular disease) onset morbidity, the clinical care of LN patients seeks to establish a balance between optimal control of inflammation and tissue injury, while limiting exposure to the side effects of immunosuppressive therapies [4]. One of the challenges to this approach is the lack of biochemical and serologic tests that accurately reflect the extent and type of renal inflammation.

Currently, monitoring of LN relies on serological biomarkers and measures of renal dysfunction (e.g., proteinuria and serum creatinine) [5]. Although elevated anti-dsDNA antibody levels and hypocomplementemia associate with disease activity in cross-sectional analyses, longitudinal studies indicate that these traditional biomarkers do not distinguish between active SLE patients with and without LN, and are inconsistent at predicting impending flares [3]. Proteinuria and other measures of renal function also falter as accurate markers of immune-mediated renal injury. As these measures reflect kidney damage, reliance on proteinuria as a marker of renal inflammation leads to delayed initiation of treatment. Conversely, it may also lead to unnecessary prolongation or premature tapering of immunosuppressive therapy due to the persistence/resolution of urinary and/or functional abnormalities that may not reflect resolution of the inciting immunologic insult. Indeed LN-associated proteinuria frequently persists for years after renal injury, normalizing in less than 50 % of patients within 2 years [6], and often a renal biopsy is the only way to distinguish between persistent activity and a chronic inactive lesion. As a result, there has been tremendous interest in the identification of biomarkers that accurately indicate the extent, type, and course of renal inflammation in LN.

Of the potential compartments that can be surveyed to identify LN-specific biomarkers, the urinary compartment may hold the most promise. Several previous studies have compared and contrasted levels of various cyto/chemokines within serum and urine samples of patients with active LN. These studies indicate that urine levels most accurately reflect renal status as compared with serum levels of these markers [7–13]. While these studies have identified some potential urinary biomarkers for diagnosis and management of LN, such as MCP-1 [7, 9, 14–16], NGAL [8, 12, 17–21], TWEAK [11, 14, 22–24], and sVCAM-1 [13, 25–27], there has been no comprehensive survey of urinary proteins to evaluate whether the above proteins constitute the best potential markers of active renal disease. Furthermore, analyses are limited on the association between urinary analyte levels and renal disease activity indices since, in the majority of studies, paired renal biopsies were not performed. In the current study, a Luminex-based proteomics approach was used to contrast levels of 128 urinary proteins found in the urine of SLE patients with active LN (ALN) and active SLE patients without LN (ANLN) to identify potential novel urinary biomarkers.

## Methods

### Subjects and data collection

Two independent cohorts were used for this study. The discovery cohort consisted of 85 active SLE patients satisfying four or more of the revised 1997 ACR classification criteria for SLE [28] and 24 age- and sex-matched healthy controls (HC). Patients were recruited from the University Health Network and Mount Sinai Hospitals. Sixty patients had ALN, confirmed by renal biopsy performed within 2 weeks of urine sampling for 88 % of patients (mean 7.5 days from sample accrual), with the remainder having active disease (score >0 on the clinical SLE Disease Activity Index-2000 (SLEDAI-2 K) components [29]) and no clinical evidence of LN. The validation cohort consisted of 33 patients with ALN (with ≥1 of the renal SLEDAI-2 K indices scoring positive and requiring changes in therapy), 16 patients with ANLN, and 30 patients with a prior history of biopsy-proven LN who were in remission with no urinary abnormalities (except one patient with fixed proteinuria (0.82 g/day) not requiring treatment).

### Measurement of urinary analyte concentrations

All urine samples were spun to remove cellular debris and frozen at –80 ° C. To avoid repeated freeze/thaws, samples were thawed once on ice, sub-aliquoted, re-frozen at –80 ° C, and then individual aliquots thawed immediately prior to use. The urinary concentrations of 128 analytes were measured by coupled bead assay (Luminex using MILLIPLEX® Map Kits (EMC Millipore Corporation), Eve Technologies Inc.) Further information regarding the sensitivity and dynamic range of the assays can be found on the company website (http://www.emdmillipore.com/). For the majority of assays, the urine samples were run undiluted except: KIM-1 and renin (diluted 1/2); albumin, beta-2-microglobulin, clusterin, cystatin C, and osteopontin (diluted 1/50); and TIMP-1 and TIMP-2 (diluted 1/5). For the discovery phase, all analytes were measured in duplicate for ALN patient samples, with a single sample on each of two separate plates, and averaged. ANLN and HC samples were measured in singles and randomly assigned to one of the two plates with equivalent numbers for each group per plate. As the duplicates run on separate plates were very reproducible, samples in the validation phase were run in singles on a single plate. All results were normalized to urinary creatinine prior to analysis.

### Renal histopathology scoring

ISN-RPS histopathological class [30] and activity and chronicity scores [31, 32] were determined by an individual renal pathologist (CA-C), blinded to the results of the urine protein determination.

### Statistical analyses of discovery data

Normalized protein data were loaded into the R statistical environment (v3.1.1) for analyses. Data were scaled using the standard deviation for each variable and hierarchical clustering performed using divisive analysis (DIANA) with a Pearson’s correlation as a similarity metric. The Adjusted Rand Index, available in the mclust package (v4.4), was used to corroborate the clustering. Normalized abundance data were correlated with clinical variables across all patients using Spearman's correlation, followed by false discovery rate (FDR) adjustment of the *p* values to correct for multiple testing. A Venn diagram was generated using the VennDiagram package (v1.6.9) to visualize the overlap of significantly correlated genes among clinical variables [33]. Data were log_2_ transformed and linear modeling was performed with the limma package (v3.20.9) in R to identify proteins with significant differences in abundance between groups. An empirical Bayes moderation of the standard error [34], followed by FDR correction, was employed [35]. Coefficients (i.e., log_2_ fold-changes relative to control samples) that were determined to be significantly different from 0 following FDR correction (*p*_adj_ < 0.01) were carried forward. A Venn diagram was generated (as described above) to visualize overlap of significantly altered proteins among clinical variables, with hypergeometic testing performed to determine if overlap was greater than expected by chance alone. Finally, results (magnitude, direction, and significance of change) were visualized in a dotmap using the lattice (v0.20-29) and latticeExtra (v0.6-26) packages for R.

A receiver operating characteristic (ROC) analysis was performed to evaluate the ability of various analytes to discriminate between active patients with or without nephritis, or with and without proliferative changes on renal biopsy. A total of 9 analytes were evaluated using data from 85 active lupus patients for the analysis of nephritis and 8 analytes on 60 patients with paired renal biopsies for analysis of proliferative nephritis. The pROC package (v1.8) for R (v3.2.1) was used to calculate the true positive (sensitivity) and false positive (1 – specificity) rates across various analyte level thresholds, along with the area under the curve (AUC). ROC curves were created using the lattice (v0.20-33) and latticeExtra (v0.6-26) packages for R.

To assess the ability of selected analytes alone or in combination with conventional biomarkers to discriminate between proliferative and non-proliferative/chronic nephritis, all possible combinations of conventional biomarkers (C3, anti-dsDNA, albuminuria) and the top performing univariate analytes associated with the activity index (vWF, IP-10, PDGF-BB, IL-16, adiponectin) were evaluated. Samples were divided into four balanced folds (such that each fold contained a similar number of active proliferative (1) and non-proliferative (0) nephritis patients). For each combination of analytes, a linear model was fit using disease status (1/0) as the response to be predicted by the indicated analytes (i.e., response ~ analyte.1 + analyte.2 …). Each model was trained using data from three of the four folds, with the fourth fold used for testing. This process was repeated four times such that each fold was used for testing only once. Mean sensitivity, specificity, and accuracy were calculated across the four repetitions.

### Statistical analysis of validation data

Statistical significance of differences between groups (e.g., ALN and ANLN) was determined using the Mann-Whitney *U* test with a Bonferroni correction for multiple comparisons.

## Results

### A cluster of urinary proteins identifies patients with active LN

**Fig. 1:**
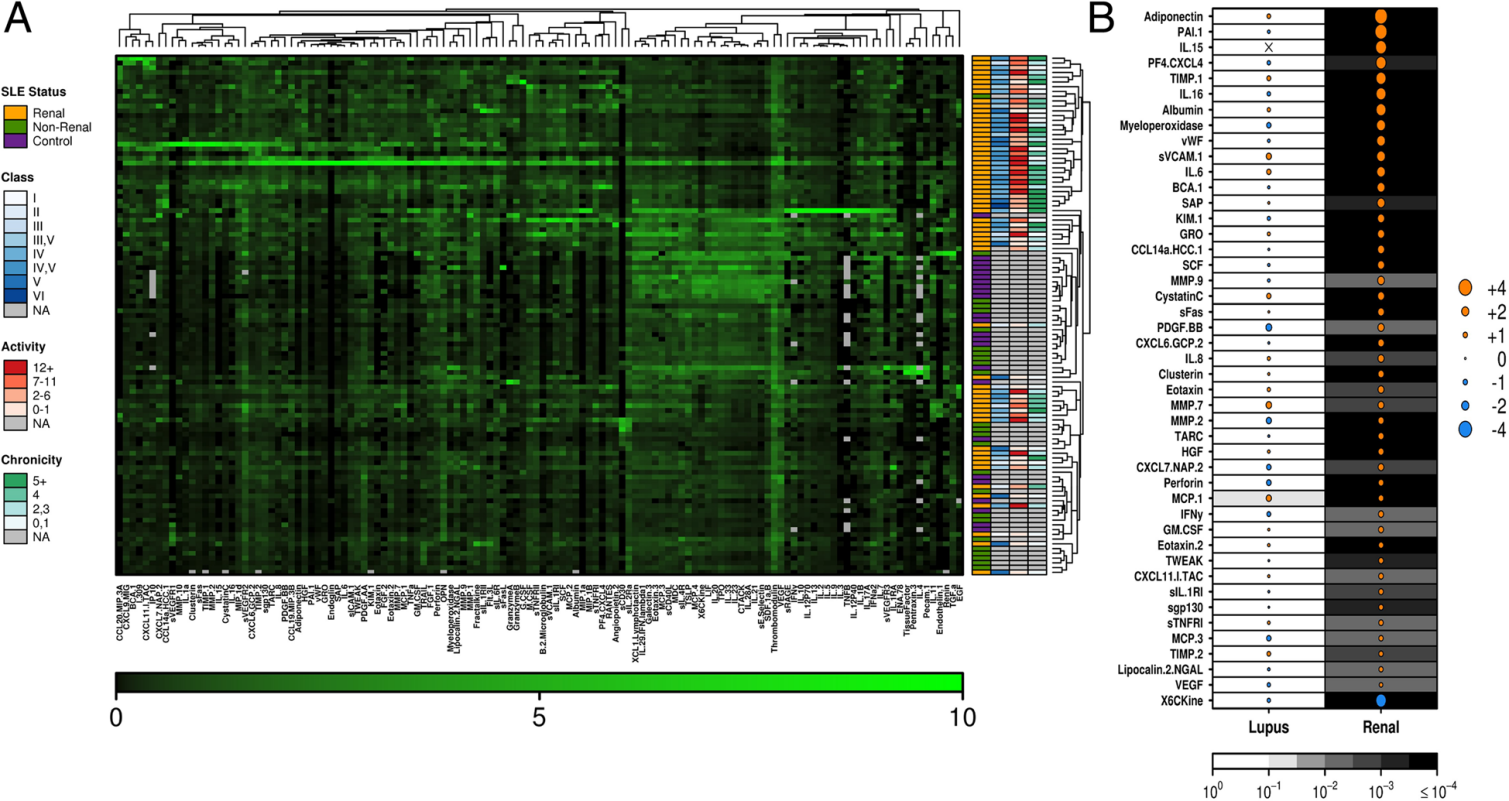
Urinary protein differences between patient groups. **a** Urinary protein levels for 85 systemic lupus erythematosus (*SLE*) patients (60 active LN, 25 active non-LN) and 24 controls in the discovery cohort. Shown are hierarchical clustering results for the 128 analytes tested as measured by Luminex. Signal intensities were adjusted using SD scaling, with *green* indicating overexpression. Hierarchical clustering was performed on the samples (rows) and proteins (columns) using divisive analysis. **b** Log_2_ fold-change of normalized protein abundance for comparisons between SLE patients and healthy controls (*left column*) and between active SLE patients with and without LN (*right column*). The size of the circles indicates the fold-change for the comparison, with *orange circles* indicating overexpression and *blue circles* indicating underexpression in all SLE patients (*left column*) or patients with LN (*right column*). The background in each row/column indicates the statistical significance of the comparison as determined by multivariate linear modeling followed by FDR correction

Urinary concentrations of 128 analytes (see Additional file 1: Table S1) were determined in SLE patients with ALN or ANLN, and HC, and normalized to urinary creatinine. Both SLE groups were comparable with respect to age, sex, and disease activity, although patients with ALN were on higher mean doses of prednisone and more immunosuppressive medications as compared with ANLN (Table 1). Hierarchical clustering (Fig. 1a) demonstrated that distinct groups of analytes identified ALN patients versus both HC and patients with ANLN (Adjusted Rand Index = 0.138, a measure of agreement between clusters with 0 indicating no agreement and 1 complete agreement), with no specific protein cluster discriminating between the latter two groups. These results suggested that a discrete number of urinary proteins preferentially identified patients with ALN.

**Table 1:** Demographic and clinical variables for SLE patients in discovery and validation cohorts

	Discovery			Validation		
Demographic variables	SLE Active LN (*n* = 60)	SLE Active non-LN (*n* = 25)	Healthy controls (*n* = 24)	SLE Active LN (*n* = 33)	SLE Active non-LN (*n* = 16)	SLE Remission LN (*n* = 30)
Age						
Range (years)	18–64	18–61	23–47	18–56	19–67	19–67
Mean ± SD (median)	35.5 ± 12.7 (32)	30.0 ± 11.9 (26)	32.8 ± 6.7 (31)	28.5 ± 9.5 (27)	36.8 ± 15.9 (36)	40.0 ± 13.6 (40.5)
Gender, n (%) female	46 (82.1)	22 (88)	21 (87.5)	28 (84.8)	15 (93.8)	28 (93.3)
Ethnicity, n (%)						
Caucasian	29 (48.3)	9 (36)	14 (58.3)	14 (42.4)	6 (37.5)	20 (66.7)
African Canadian	14 (23.3)	7 (28)	1 (4.2)	8 (24.2)	2 (12.5)	4 (13.3)
Asian	9 (15)	5 (20)	3 (12.5)	5 (11.6)	2 (12.5)	2 (6.7)
Other^a^	8 (13.3)	4 (16)	6 (25)	6 (18.2)	6 (37.5)	4 (13.3)
Clinical features^b^, n (%)						
Rash	8 (14.3)	12 (48)	N/A	11 (33.3)	3 (18.8)	0
Mucocutaneous	9 (16.1)	9 (36)	N/A	0	2 (12.5)	0
Alopecia	4 (7.1)	7 (28)	N/A	4 (12.1)	5 (31.2)	1 (3.3)
Arthritis	13 (23.2)	13 (52)	N/A	3 (9.1)	9 (56.2)	0
Serositis	8 (14.3)	4 (16)	N/A	2 (6.1)	1 (6.2)	0
Myositis	1 (1.8)	0	N/A	0	1 (6.2)	0
Hematological	6 (10.7)	6 (24)	N/A	2 (6.1)	3 (18.8)	0
Fever	3 (5.4)	1 (4)	N/A	1 (3.0)	2 (12.5)	0
Nephritis (≥1 or more SLEDAI-2 K criteria)	59 (98.3)^c^	0	N/A	33 (100)	0	1 (3.3)
Vasculitis	3 (5.4)	7 (28)	N/A	2 (6.1)	3 (18.8)	0
SLEDAI-2 K, mean ± SD (median)	15.6 ± 7.4 (16)	10.1 ± 4.7 (10)	N/A	14.2 ± 5.6 (14)	7.2 ± 2.7 (8)	1.7 ± 1.9 (1)
Creatinine, mean ± SD (median)	118.0 ± 78.8 (87)	64.5 ± 9.6 (62)	N/A	91.8 ± 81.1 (69)	69.2 ± 16.0 (70.5)	81.8 ± 25.3 (75.5)
Medications^b^						
Prednisone (mg/day), mean ± SD (median)	28.5 ± 20.8 (30)	10.8 ± 30.4 (0)	N/A	21.8 ± 18.8 (15)	8.5 ± 13.2 (4)	6.1 ± 6.9 (4)
Anti-malarials, n (%)	31 (55.4)	15 (60)	N/A	21 (63.6)	13 (81.2)	24 (80)
Immunosuppressives, n (%)	55 (91.7)	5 (20)	N/A	27 (81.8)	10 (62.5)	22 (73.3)
Azathioprine, n (%)	9 (16.1)	3 (12)	N/A	11 (33.3)	3 (18.8)	9 (30)
Mycophenolate mofetil, n (%)	15 (26.8)	1 (4)	N/A	9 (27.3)	2 (12.5)	10 (33.3)
Methotrexate, n (%)	1 (1.8)	1 (4)	N/A	1 (3.0)	5 (31.2)	1 (3.3)

In the validation cohort, eight patients had renal biopsies an average of 10 days from sample accrual

^a^Other ethnicities include Hispanic, Filipino, mixed, and, for three healthy controls, unknown ethnicity

^b^Clinical variables and treatment at time of recruitment

^c^One patient had a renal biopsy performed for 0.45 g/24 h proteinuria

*LN* lupus nephritis, *N/A* not applicable, *SD* standard deviation, *SLE* systemic lupus erythematosus, *SLEDAI-2 K* Systemic Lupus Erythematosus Disease Activity Index-2000

**Fig. 2:**
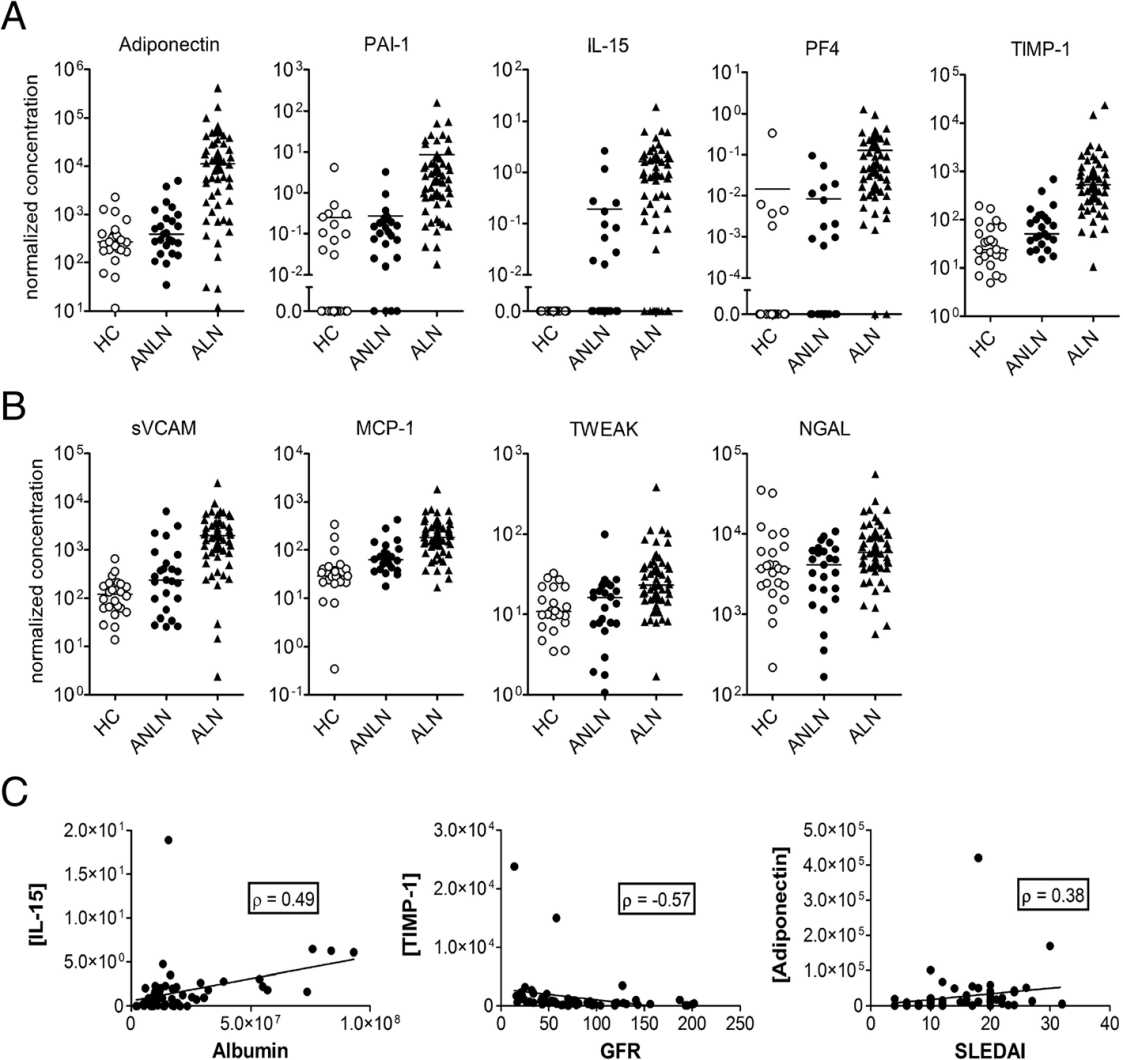
Comparison of the most highly discriminative urinary analytes to several previously proposed biomarkers. Scatter plots showing the normalized concentration of selected urinary proteins in healthy controls (*HC*; *n* = 24, *open circles*) and SLE patients with active non-LN (*ANLN*; *n* = 25, *closed circles*) or active LN (*ALN*; *n* = 60, *closed triangles*). Urinary concentrations were corrected for creatinine to normalize for osmolality. Units for all graphs are pg/μmol, except PF4 and TWEAK which are in ng/μmol. **a** Urinary analytes that demonstrated >3 log_2_ fold-difference between ANLN and ALN. **b** Previously proposed urinary biomarkers. For all graphs each symbol represents the determination from a single individual, with the mean value for each group indicated by a *horizontal line*. Fold-differences and statistical comparisons for the analytes are shown in Table 2. **c** Correlation between the urinary analytes that demonstrated >3 log_2_ fold-difference between ANLN and ALN and that showed the strongest association with albuminuria, estimated glomerular filtration rate (*GFR*), or Systemic Lupus Erythematosus Disease Activity Index-2000 (*SLEDAI*). *Lines* indicate linear regression curves. Spearman’s correlation coefficients are shown

To explore the relationship between individual urinary analytes and the three clinical states, linear modeling was performed. No statistically significant difference in urinary protein expression was seen when comparing all SLE patients (ALN and ANLN) to HC (Fig. 1b, left column). In contrast, statistically significant differences in analyte levels (*p*_adj_ < 0.01) were noted between the two active SLE populations with 44 proteins preferentially elevated in patients with ALN (Fig. 1b, right column, and Table 2). Differences between the two patient populations were striking, with 13 proteins having >4-fold (2 log_2_ fold-change in the table) difference in the mean urinary concentration between patients with ALN versus ANLN, five of which (adiponectin, PAI-1, IL-15, PF4, and TIMP-1) demonstrated >8-fold increase in abundance (Fig. 2a). The concentration of these analytes in patients with ANLN was statistically indistinguishable from levels detected in HC, supporting their candidacy as LN-specific biomarkers. In further support of the concept that these biomarkers are specific for active renal disease in SLE, there was no association between the SLEDAI and urinary analyte levels in ANLN patients, and any significant associations between the SLEDAI and urinary analyte levels in the total patient cohort (or ALN subset) were lost when the renal components of the SLEDAI were removed from the calculation.

**Table 2:** Results of statistical analysis of discovery and validation cohorts

	Discovery cohort		Validation cohort	
Urinary analyte	Log_2_ fold-difference ALN vs ANLN	*p*_adj_*	*p*_adj_* ALN vs ANLN	*p*_adj_* ALN vs RLN
Adiponectin	4	≤0.0001	≤0.0044	≤0.0018
PAI-1	3.77	≤0.0001	≤0.0044	≤0.0018
IL-15	3.32	≤0.0001	≤0.0044	≤0.0018
PF4 (CXCL4)	3.04	≤0.0001	≤0.0044	≤0.0018
TIMP-1	3.01	≤0.0001	≤0.0044	≤0.0018
IL.16	2.97	≤0.0001	0.62	N/A
Albumin	2.95	≤0.0001	0.022	1
MPO	2.62	≤0.0001	0.81	N/A
vWF	2.5	≤0.0001	≤0.0044	≤0.0018
sVCAM-1	2.49	≤0.0001	≤0.0044	≤0.0018
IL-6	2.46	≤0.0001	0.0088	≤0.0018
BCA-1	2.22	≤0.0001	0.54	N/A
SAP	2.19	0.001	0.33	N/A
KIM-1	1.95	≤0.0001	0.044	0.013
GRO	1.93	≤0.0001	≤0.0044	0.0054
HCC-1 (CCL14a)	1.91	≤0.0001	ND	N/A
SCF	1.89	≤0.0001	1	N/A
MMP-9	1.89	0.007	1	N/A
Cystatin C	1.87	≤0.0001	0.044	0.0072
sFas	1.76	≤0.0001	0.18	N/A
PDGF-BB	1.7	0.005	0.80	N/A
GCP-2 (CXCL6)	1.69	≤0.0001	≤0.0044	≤0.0018
IL-8	1.66	0.002	0.0088	0.26
Clusterin	1.58	≤0.0001	≤0.0044	≤0.0018
Eotaxin	1.58	0.002	1	N/A
MMP-7	1.56	0.002	0.022	≤0.0018
MMP-2	1.56	≤0.0001	1	N/A
TARC	1.48	≤0.0001	1	N/A
HGF	1.47	≤0.0001	0.44	N/A
NAP-2 (CXCL7)	1.47	0.001	1	N/A
Perforin	1.37	≤0.0001	1	N/A
MCP-1	1.34	≤0.0001	≤0.0044	≤0.0018
IFNγ	1.29	0.005	1	N/A
GM-CSF	1.24	0.007	0.0088	0.013
Eotaxin-2	1.22	≤0.0001	1	N/A
TWEAK	1.16	0.001	0.54	N/A
I-TAC (CXCL11)	1.13	0.007	0.079	N/A
sIL-1RI	1.12	0.003	1	N/A
sgp130	1.07	≤0.0001	1	N/A
sTNFRI	1.06	0.004	1	N/A
MCP-3	1.02	0.006	1	N/A
TIMP-2	1.01	0.002	1	N/A
NGAL	0.95	0.01	ND	N/A
VEGF	0.72	0.007	1	N/A
X6CKine	–3.15	≤0.0001	1	N/A

*p values have been adjusted for multiple testing to reduce the false discovery rate

*N/A* not applicable, *ND* not determined

### Novel candidate urinary biomarkers outperform currently proposed LN activity-specific biomarkers

MCP-1, TWEAK, NGAL, and sVCAM have been proposed as potential LN activity-specific biomarkers in adult and pediatric SLE patients [7–9, 11–23, 25–27]. Our findings confirmed that these urinary analytes are elevated in the urine of ALN as compared to ANLN patients (Fig. 2b and Table 2). However, the differences between the two patient populations were not as marked as those noted for many of our newly identified urinary analytes. These results suggest that our newly identified proteins may have a greater capacity to discriminate ALN from ANLN. To assess this possibility we contrasted the receiver operating characteristics (ROC) for the five analytes with the highest fold-difference between ALN and ANLN, with previously proposed urinary and serum (C3, anti-dsDNA) biomarkers, as well as established markers of renal dysfunction (Table 3; see Additional file 1: Figure S1). With the exception of sVCAM, the AUCs were higher and the sensitivities and specificities at optimal cut-offs were improved as compared to existing biomarkers. Furthermore, for many of the analytes, the AUC and sensitivities approached those seen for albuminuria, with several of the analytes demonstrating improved specificity over this conventional measure.

**Table 3:** Results of the ROC analysis for diagnosis of ALN

Analyte	Sensitivity	Specificity	AUC	Best cut-off
Adiponectin	0.77	0.92	0.88	1.86 × 10^3^
PAI-1	0.82	0.92	0.90	4.47 × 10^–1^
IL-15	0.70	0.96	0.84	3.05 × 10^–1^
PF4	0.93	0.76	0.90	2.44 × 10^–3^
TIMP-1	0.90	0.84	0.91	1.26 × 10^2^
sVCAM	0.83	0.76	0.81	5.29 × 10^2^
MCP-1	0.75	0.84	0.81	1.25 × 10^2^
TWEAK	0.47	0.96	0.73	2.73 × 10^–2^
NGAL	0.85	0.48	0.69	3.35 × 10^3^
Albumin	0.95	0.84	0.93	4.90 × 10^6^
Creatinine	0.58	0.96	0.76	8.05 × 10^1^
C3	0.57	0.72	0.57	7.60 × 10^–1^
anti-dsDNA	0.30	0.80	0.48	1.00 × 10^2^

*ALN* active lupus with lupus nephritis, *AUC* area under the curve, *ROC* receiver operating characteristic

Notably, within the subset of patients with ALN, there was a poor correlation between the levels of these analytes and albuminuria (ρ ranging from 0.14 for TIMP-1 to 0.49 for IL-15), eGFR (ρ ranging from –0.04 for adiponectin to –0.57 for TIMP-1) or SLEDAI (ρ ranging from 0.16 for TIMP-1 to 0.38 for adiponectin). Representative plots showing the analytes with the strongest correlation for each of these parameters are shown in Fig. 2c.

### A subset of urinary biomarkers correlates with the presence of active proliferative renal lesions on histopathology

As all of the ALN patients in the discovery cohort had a renal biopsy performed close to the time of their urine sampling (see Additional file 1; biopsy characteristics summarized in Additional file 1: Table S2), a Spearman correlation analysis followed by correction for multiple testing was performed to further delineate the relationship between urinary analytes and renal disease activity. Ten proteins (adiponectin, PAI-1, IL-16, wVF, IP-10, TIMP-1, eotaxin, sgp130, HGF, and PDGF-BB) were found to significantly correlate with renal biopsy activity score (q < 0.01; see Additional file 1: Table S3), with no analytes correlating with chronicity scores. Of the individual components of the activity index, only glomerular hyaline thrombi/wire loops and interstitial monocytes were not independently associated with these urinary biomarkers, with the strongest association being with the cellular crescents sub-score (ρ = 0.39 to 0.63) for the majority of the biomarkers.

**Fig. 3:**
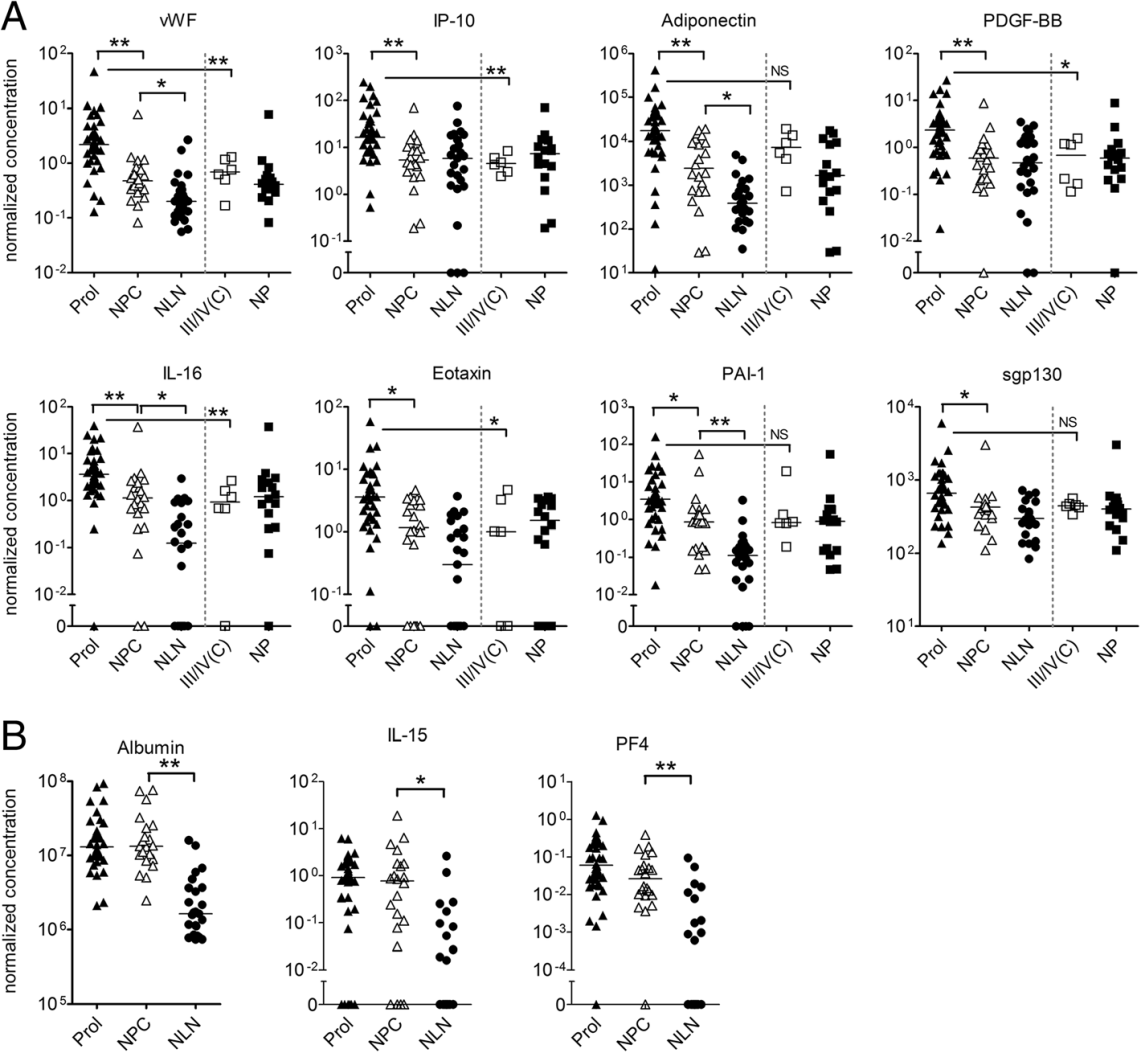
Urinary proteins that discriminate between proliferative and non-proliferative/chronic lesions on renal biopsy. Scatter plots showing the normalized concentration of selected urinary proteins in SLE patients with proliferative LN (*Prol*; Class III and IV (A) or (A/C), *n* = 38, *closed triangles*) and non-proliferative or chronic lesions (*NPC*; Class I, II, V, or VI, and Class III or IV (C), *n* = 22, *open triangles*) on renal biopsy as compared to active SLE patients without LN (*NLN*; *n* = 25, *closed circles*). Units for all analytes are pg/μmol, except vWF and PF4 which are in ng/μmol. **a** Plots for the eight urinary proteins that discriminated between proliferative and NPC lesions on renal biopsy. In the two columns on the right of each plot the NPC results have been subdivided into chronic class III/IV (*III/IV(C)*) and other non-proliferative/chronic lesions (*NP*). **b** Plots for three representative urinary proteins that did not discriminate between proliferative and non-proliferative or chronic lesions on renal biopsy, demonstrating their ability to discriminate between non-proliferative/chronic lesions and active non-LN. For all graphs, each symbol represents the determination from a single individual, with the mean value for each group indicated by a *horizontal line*. Significance levels were determined by Mann-Whitney non-parametric testing and have been corrected for multiple testing. Only the differences between proliferative and non-proliferative/chronic lesions, and non-proliferative/chronic lesions and active non-LN for each analyte are shown. In graphs in panel (a), significant differences between active proliferative and chronic class III/IV lesions are also indicated, and represent uncorrected *p* values. *p < 0.05, **p <0.005, *NS* not significant

Given that the activity score is driven primarily by histopathological features associated with proliferative lesions [31, 32], we examined whether any of these activity index-associated proteins discriminated between active proliferative (ISN/RPS III (A or A/C), IV (A or A/C)) and other renal lesions including non-proliferative (ISN/RPS I, II, V) or chronic lesions (ISN/RPS III (C), IV (C), V (C) or VI). As shown in Fig. 3a, eight of these proteins were found to significantly discriminate between proliferative and non-proliferative or chronic lesions (see Additional file 1; ROC analysis shown in Additional file 1: Table S4), and similar findings were observed when just the subset of class III/IV patients with chronic lesions was examined. For four of these analytes, the increases detected in ALN were driven solely by patients with active proliferative lesions, indicating that some urinary biomarkers may be specific for proliferative lesions. In contrast, urinary elevations of albumin, IL-15, and PF4 did not differentiate between proliferative and non-proliferative/chronic lesions on biopsy, but appeared to effectively discriminate patients with non-proliferative/chronic lesions from patients without LN (Fig. 3b). Notably, none of the urinary analytes discriminated between class II or V and chronic renal lesions, nor were there urinary analytes that could discriminate between class III and IV LN. There were no significant differences in the levels of the urinary analytes between class III/IV patients with or without class V changes. Although there were trends to higher levels of the activity-correlated urinary proteins in class IV as compared to class III nephritis and in class IV-G as compared to class IV-S nephritis, these did not achieve statistical significance when corrected for multiple comparisons. There was no association between corticosteroid dose at the time of urine sampling and the levels of any of the urinary analytes.

To investigate whether measurement of urinary analytes associated with the activity index results in an improved ability to discriminate between active proliferative and non-proliferative or chronic nephritis over conventional biomarkers, such as anti-dsDNA, C3, and albuminuria, we performed a multivariate analysis assessing all combinations of these conventional biomarkers together with the top five performing urinary analytes from the univariate analysis. The most accurate combination of conventional biomarkers was C3 and dsDNA (accuracy of 0.7675, sensitivity 0.875, specificity 0.5925). A number of different combinations of two to four urinary analytes together with anti-dsDNA had improved specificities and accuracies over this combination (see Additional file 1: Table S5), with the top performing combination being anti-dsDNA + vWF + IP-10 + PDBG-BB + adiponectin (accuracy 0.8325, sensitivity 0.87, specificity 0.7675). These findings support the potential clinical utility of measurement of these activity-specific biomarkers in the diagnosis of active proliferative nephritis.

### Validation of potential urinary biomarkers in a secondary cohort and normalization in renal remission

**Fig. 4:**
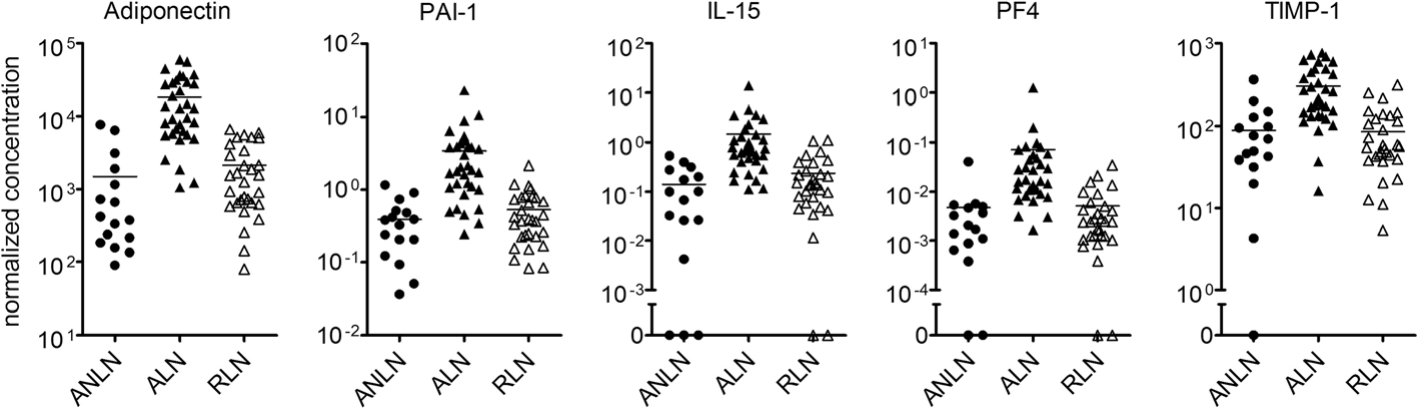
Normalization of urinary protein levels in LN patients in remission. Scatter plots showing the normalized concentration of selected urinary proteins in SLE patients from the validation cohort with active non-LN (*ANLN*; *n* = 16, *closed circles*), active LN (*ALN*; *n* = 33, *closed triangles*) or LN in remission (*RLN*; *n* = 30, *open triangles*). Units for all graphs are pg/μmol, except for PF4 which is in ng/μmol. Each symbol represents the determination from a single individual, with the mean value for each group indicated by a *horizontal line*. Statistical comparisons for the analytes are shown in Table 2

To validate the findings in the discovery cohort, we measured the levels of the significantly increased urinary analytes from this cohort in a second independent cohort of 33 ALN and 16 ANLN SLE patients (see demographics in Table 1). In this smaller cohort, 18 of the original analytes identified replicated, including all five of the urinary analytes that previously demonstrated >3 log_2_ fold-increase in LN (Table 2 and Fig. 4).

A key requisite of an activity-specific LN biomarker is normalization during renal remission. Therefore the levels of the replicated urinary LN biomarkers in the ALN patients were contrasted with those in 30 patients with previous LN that were in remission. Of the 18 replicated urinary analytes, all but two (albumin and IL-8) normalized in patients in remission (Table 2 and Fig. 4). These findings support the potential clinical utility of these analytes as biomarkers of active renal involvement in SLE.

## Discussion

In this study we used an unbiased proteomics-based approach to compare the levels of 128 proteins in the urine of active SLE patients with and without LN. We show that many urinary proteins are elevated in patients with ALN, with approximately one-third of the analytes tested being at least 2-fold increased in the urine of patients with ALN as compared to ANLN. These proteins represent a broad array of molecules, many of which have been implicated in the pathogenesis of nephritis through diverse mechanisms including: cyto/chemokines and their receptors (e.g., IL-15 and PF4), metalloproteinases and their regulators (e.g., TIMP-1 and MMP-9), growth factors (e.g., GM-CSF), markers of endothelial injury/repair (e.g., adiponectin, PAI-1, and vWF), and markers of kidney damage (e.g., KIM-1 and cystatin C).

Although elevated levels of multiple proteins were seen in the urine of patients with ALN, these elevations did not appear to result solely from the decreased filtering capacity of the kidney in LN. For those urinary analytes that demonstrated >4-fold increase in the urine of ALN patients, there was no or only a weak correlation with albuminuria (Fig. 2c, and data not shown). Furthermore, consistent with previously published reports [7–13], we have previously observed a poor correlation between serum and urine levels of MCP-1, adiponectin, and sVCAM-1 (unpublished observations). These findings suggest that the elevated levels of urinary proteins in LN reflect increased elaboration within the kidney as a consequence of active inflammation. In support of this concept, increased kidney expression of many of the proteins identified in the current study has been described in various nephritis models [10, 13, 36–39].

A highlight of our study was the identification of a number of biomarkers for ALN with markedly elevated urinary levels as compared to ANLN and remission LN (>4–8-fold). Of the nine urinary proteins in this group that replicated in the validation cohort, four have been previously reported to be elevated in ALN. These include adiponectin [10], albumin [40], sVCAM-1 [13, 25–27], and IL-6 [41, 42]. The remaining five, including PAI-1, IL-15, PF4, TIMP-1, and vWF, represent novel potential urinary biomarkers that have not previously been described in LN. Notably, a number of these biomarkers appeared to outperform previously proposed biomarkers such as NGAL, MCP-1, and TWEAK, with improved sensitivities and specificities at optimal cut-offs. These differences do not appear to reflect differences in the ability of the Luminex system to detect these previously reported biomarkers, because the fold-increases and sensitivity and specificity at optimal cut-offs were very similar in our study to those observed in previous studies [7, 11, 15, 18, 23].

While several of the biomarkers that demonstrated the greatest fold-differences between ALN and ANLN demonstrated very high sensitivities (up to 93 %) and specificities (up to 96 %), no one biomarker appeared to be sufficient to diagnose ALN with 100 % accuracy, and it is likely that a panel of biomarkers may more optimally discriminate LN from other disease states. Based upon our ROC results, it is probable that a panel consisting of a combination of a subset of the five analytes demonstrating >8-fold difference between ALN and ANLN would offer the best discriminative ability; however, this will require testing in an independent unselected cohort of SLE patients with both active and inactive disease.

Previous work has shown that renal biopsies demonstrating diffuse proliferative (class IV) LN and increased glomerular activity indices are associated with a poorer long-term prognosis [31, 32, 43, 44], an increased likelihood of re-flare [45], and increased chronicity scores on subsequent renal biopsies with glomerulosclerosis and consequent renal functional deterioration [46]. Given the invasive nature of renal biopsies, there has been considerable interest in the identification of urinary biomarkers that accurately reflect renal disease activity scores and/or proliferative nephritis. In this study, we identified 10 urinary analytes that showed a strong correlation with the renal biopsy activity score. Although several urinary proteins, including sVCAM [47], NGAL [8, 48], MCP-1 [48, 49], adiponectin [48], KIM-1 [48], and TWEAK [24], have been previously reported to correlate with the activity index on renal biopsy, with the exception of adiponectin none of these analytes were replicated in our study. Thus, we have identified nine novel proteins that are more closely associated with the activity index on renal biopsy than all previously reported associated proteins except adiponectin. Notably, addition of various combinations of the five top performing analytes associated with the activity index to conventional biomarkers of renal disease activity (C3, anti-dsDNA, and albuminuria) resulted in an improved ability to discriminate between active proliferative and non-proliferative or chronic nephritis, supporting their potential clinical utility for the diagnosis of active proliferative nephritis.

Recently a composite urinary biomarker activity index was reported based upon the urinary levels of six proteins, including adiponectin, NGAL, MCP-1, KIM-1, ceruloplasmin, and hemopexin, that can differentiate pediatric LN patients with high activity indices on renal biopsy from those with low or moderate activity indices with >92 % accuracy [48]. Our findings raise the possibility that further improvements in this accuracy might be possible through the use of alternate biomarkers that are more closely associated with renal disease activity, a possibility that can be addressed in further studies.

Given the large number of urinary analytes examined in our discovery cohort, it was important to replicate these findings. Although only 18 of the biomarkers examined were replicated, it is likely that this results from several differences between our discovery and validation cohorts. Firstly, our validation cohort was significantly smaller in size (approximately half the size of our discovery cohort) which may have restricted our ability to detect less robust biomarkers. Additionally, the majority of patients in our validation cohort with ALN were re-flaring, usually after several years of treatment, whereas the majority of the patients in the discovery cohort had new-onset LN. As previous work suggests that repeat flares are associated with an increased likelihood of chronic rather than active changes [50], this might have affected our ability to replicate urinary analytes that are associated predominantly with active proliferative lesions, such as IL-16, PDGF-BB, sgp130, and eotaxin. Finally, there may have been significant differences between the cohorts in the distribution of renal biopsy classes (e.g., more class V in the validation cohort), which may also have affected our ability to replicate these differences. If the inability to validate these activity-associated analytes is related to these cohort differences, then these biomarkers may still be clinically useful as biomarkers that are specific for active class III/IV LN.

The majority of the validated urinary analytes were found to normalize in patients who had achieved renal remissions. These findings suggest that measurement of these urinary proteins will have clinical utility for monitoring responses to therapy and prediction of flare, as has been shown for some of the previously published urinary biomarkers, such as NGAL [12, 18–21] and TWEAK [23]. Longitudinal studies are ongoing to define which combination of the previously published biomarkers and novel biomarkers identified in this study best reflects changes in LN activity and response to therapy, with the goal of defining an optimal biomarker panel for the monitoring of LN.

## Conclusions

Using a proteomics approach, novel urinary biomarkers were identified and validated that discriminate between active SLE patients with and without nephritis, and normalize in patients in remission. Several of these novel urinary proteins offer improved discriminative ability over many previously identified urinary biomarkers. A subset of these urinary proteins effectively discriminated between active proliferative and non-proliferative/chronic lesions in paired renal biopsies, suggesting that they may have clinical utility in the identification of patients that may require more aggressive therapy.

## Additional file

Additional file 1: Supplementary data. Supplementary information to the results. (DOCX 1249 kb)

## Abbreviations

ALN: Active lupus with lupus nephritis
ANLN: Active lupus without lupus nephritis
AUC: Area under curve
FDR: False discovery rate
HC: Healthy control
LN: Lupus nephritis
ROC: Receiver operating characteristic
SLE: Systemic lupus erythematosus
SLEDAI-2 K: Systemic Lupus Erythematosus Disease Activity Index-2000
