# Supplementary material for: A discrete cluster of urinary biomarkers discriminates between active systemic lupus erythematosus patients with and without glomerulonephritis
Source: Arthritis Res Ther. 2016 Oct 4;18:218. doi: 10.1186/s13075-016-1120-0 (PMC5050957; doi:10.1186/s13075-016-1120-0)
Supplement: Additional file 1: — Supplementary data. Supplementary information to the results. (DOCX 1249 kb) [file 13075_2016_1120_MOESM1_ESM.docx]

**Additional file 1**

**Table S1.** List of urinary analytes tested in the discovery cohort.

| **Urinary Analytes Tested (n = 128)** | | | |
| --- | --- | --- | --- |
| Adiponectin | IL-2 | MCP-2 | sgp130 |
| Albumin | IL-3 | MCP-3 | sICAM-1 |
| Angiopoietin-2 | IL-4 | MCP-4 | sIL-R1 |
| BCA-1/CXCL13 | IL-5 | MDC (CCL22) | sIL-RII |
| beta-2-microglobulin | IL-6 | MIG/CXCL9 | sIL-2Ra |
| Clusterin | IL-7 | MIP-1α | sIL-4R |
| CTACK/CCL27 | IL-8 | MIP-1β | sIL-6R |
| Cystatin C | IL-9 | MIP-1d (MIP-5/CCL15) | sRAGE |
| EGF | IL-10 | MIP-3a/CCL20 | sTNF RI |
| Endoglin | IL-11 | MIP-3b/CCL19 | sTNF RII |
| Endothelin-1 | IL-12 (p40) | MMP-1 | sVCAM-1 |
| Eotaxin | IL-12 (p70) | MMP-2 | sVEGF R1 |
| Eotaxin-2/CCL24 | IL-13 | MMP-7 | sVEGF R2 |
| Eotaxin-3/CCL26 | IL-15 | MMP-9 | sVEGF R3 |
| ENA-78/CXCL5 | IL-16 | MMP-10 | SCF |
| FGF-1 | IL-17 | MPO | SDF-1 α+β/CxCL12 |
| FGF-2 | IL-20 | NAP-2/CXCL7 | Serum Amyloid P |
| Flt-3 ligand | IL-21 | Osteopontin | TARC/CCL17 |
| Fractalkine | IL-23 | PDGF-AA | TGF-α |
| Galectin 3 | IL-28a | PDGF-AB/BB | Thrombomodulin |
| G-CSF | IL-29/IFNg2 | RANTES | TIMP-1 |
| GM-CSF | IL-33/NF-HEV (mature) | PAI-total | TIMP-2 |
| Granzyme A | I-309 | Pecam-1 | Tissue Factor (TF) |
| Granzyme B | IP-10 | Perforin | TNF-α |
| GRO | I-TAC/CXCL11 | Pentraxin-3 (PTX3) | TNF-β |
| HCC-1/CCL14a | KIM-1 | Platelet Factor 4 (PF4) | TPO |
| HGF | LIF/CCL1 | Renin | TRAIL |
| IFN-α2 | Lipocalin-2/NGAL | sCD40L | TSLP |
| IFN-γ | LIX/CXCL6 | sCD30 | TWEAK |
| IL-1α | Lymphotactin | sE-Selectin | VEGF |
| IL-1β | M-CSF | sFas | Von Willibrand Factor (vWF) |
| IL-1Rα | MCP-1 | sFasL | 6Ckine/CCL21 |

**Table S2.** Biopsy characteristics for patients with paired urine and renal biopsies in the discovery cohort

| **Biopsy Classification (ISN/RPS) n (%)** | | |
| --- | --- | --- |
|  | I | 1 (1.7) |
|  | II | 3 (5.0) |
|  | III (III/V)* | 12 (20.0) |
|  | IV (IV/V)* | 32 (53.3) |
|  | V | 9 (15) |
|  | VI | 2 (3.3) |
|  | Other (TIN) | 1 (1.7) |
| **Activity Score** | | |
|  | Range (median) | 0-19 (6) |
|  | Mean ± SD | 6.68 ± 5.57 |
| **Chronicity Score** | | |
|  | Range (median) | 0-10 (3) |
|  | Mean ± SD | 3.13 ± 2.43 |

*66.6% of class III and 37.5% of class IV biopsies had class V changes

**Table S3.** Summary statistics for urinary analytes associated with activity score on biopsy

| **Analyte** | **Correlation Coefficient (ρ)** | **P_adj_*** |
| --- | --- | --- |
| vWF | 0.65 | 3.08E-06 |
| IL-16 | 0.61 | 1.25E-05 |
| PDGF-BB | 0.53 | 0.00067 |
| IP-10 | 0.50 | 0.00128 |
| Adiponectin | 0.48 | 0.00242 |
| PAI-1 | 0.46 | 0.00347 |
| sgp130 | 0.46 | 0.00347 |
| Eotaxin | 0.47 | 0.00347 |
| TIMP-1 | 0.46 | 0.00374 |
| HGF | 0.44 | 0.00645 |

* p values have been adjusted for multiple testing to reduce the false discovery rate

**Table S4.** Results of the ROC analysis for diagnosis of proliferative LN.

| **Analyte** | **Sensitivity** | **Specificity** | **AUC** | **Best Cut-off** |
| --- | --- | --- | --- | --- |
| Adiponectin | 0.71 | 0.77 | 0.80 | 1.00 x 10^4^ |
| PAI-1 | 0.74 | 0.86 | 0.77 | 1.95 |
| IL-16 | 0.79 | 0.73 | 0.80 | 1.95 |
| vWF | 0.76 | 0.95 | 0.86 | 1.34 |
| IP-10 | 0.79 | 0.73 | 0.81 | 8.67 |
| Eotaxin | 0.53 | 0.95 | 0.76 | 3.59 |
| spg130 | 0.55 | 0.95 | 0.75 | 6.00 x 10^2^ |
| PDGF-BB | 0.71 | 0.86 | 0.80 | 1.25 |
| Albumin | 0.95 | 0.14 | 0.49 | 5.61 x 10^7^ |
| Creatinine | 0.45 | 0.68 | 0.47 | 7.85 x 10^1^ |
| C3 | 0.71 | 0.86 | 0.79 | 0.68 |
| anti-dsDNA | 0.82 | 0.59 | 0.72 | 11.5 |

**Table S5.** Results of the multivariate analysis examining the optimal combination of conventional and novel biomarkers for the diagnosis of proliferative LN.

| **Sensitivity** | **Specificity** | **Accuracy*** | **Analyte** |
| --- | --- | --- | --- |
| 0.87 | 0.7675 | 0.8325 | anti-dsDNA + vWF + IP-10 + PDGF-BB + Adiponectin |
| 0.895 | 0.675 | 0.8125 | anti-dsDNA + IP-10 + PDGF-BB |
| 0.8675 | 0.725 | 0.8125 | anti-dsDNA + vWF + IP-10 + PDGF-BB |
| 0.895 | 0.635 | 0.8 | anti-dsDNA + IP-10 + Adiponectin |
| 0.895 | 0.635 | 0.8 | anti-dsDNA + vWF + IP-10 + Adiponectin |
| 0.8675 | 0.675 | 0.795 | anti-dsDNA + IP-10 + PDGF-BB + Adiponectin |
| 0.845 | 0.685 | 0.7825 | anti-dsDNA + vWF + PDGF-BB + Adiponectin |
| 0.8175 | 0.725 | 0.7825 | anti-dsDNA + vWF + IP-10 + PDGF-BB + IL-16 + Adiponectin |
| 0.895 | 0.5925 | 0.78 | anti-dsDNA + PDGF-BB + Adiponectin |
| 0.925 | 0.525 | 0.77 | vWF + IP-10 + PDGF-BB + Adiponectin |
| 0.875 | 0.5925 | 0.7675 | C3 + anti-dsDNA |
| 0.8475 | 0.41 | 0.6825 | C3 |
| 0.77 | 0.475 | 0.66 | C3 + anti-dsDNA + Albumin |
| 0.815 | 0.3325 | 0.6325 | anti-dsDNA |
| 0.7425 | 0.3525 | 0.5975 | C3 + Albumin |
| 0.895 | 0.035 | 0.5825 | Albumin |
| 0.77 | 0.2075 | 0.5625 | anti-dsDNA + Albumin |

*results for the top 10 combinations are shown together with combinations of conventional biomarkers

**
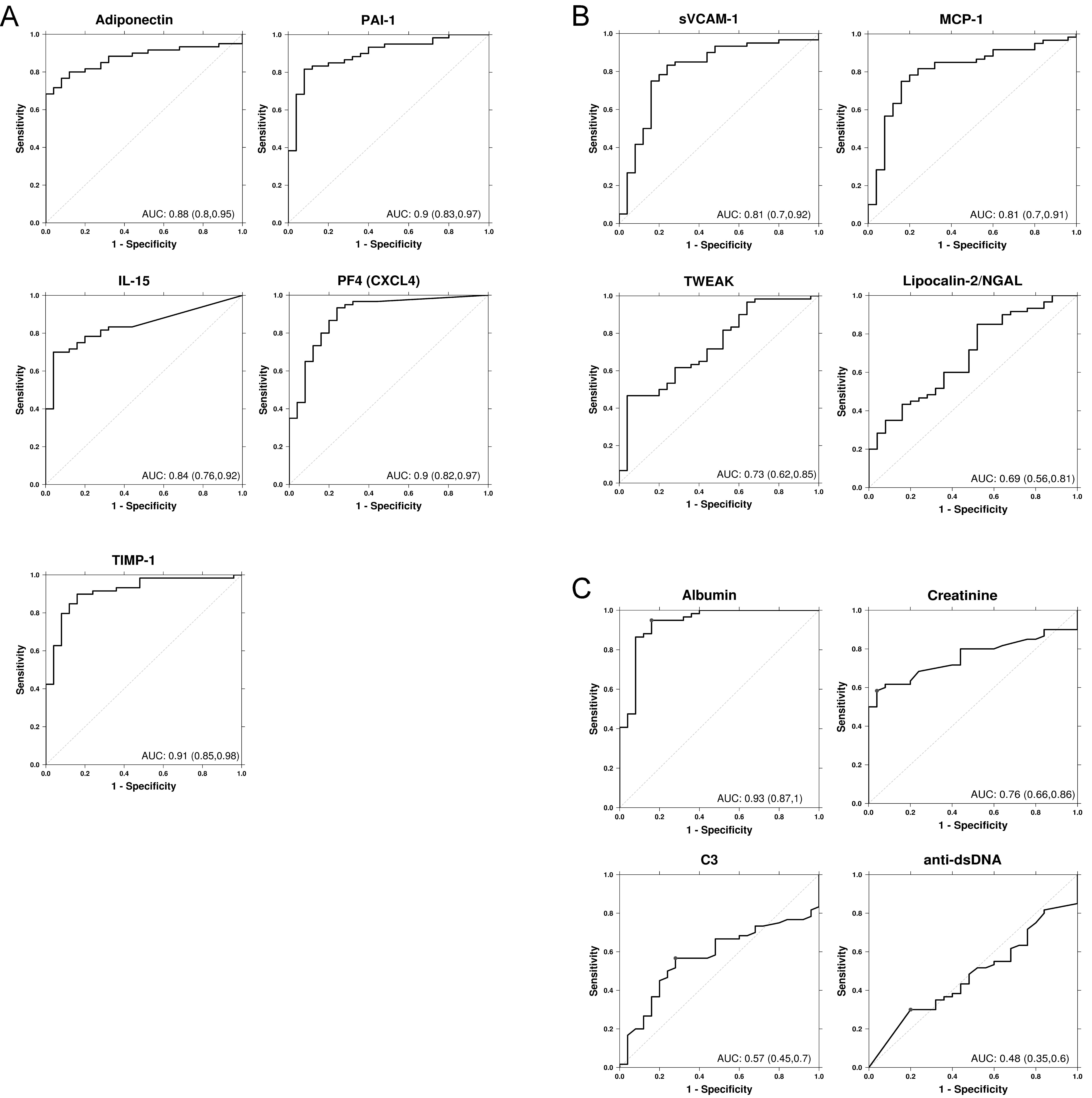
**

**Figure S1.** ROC curves for selected analytes for the diagnosis of LN. A) Urinary analytes that demonstrated a > 3 log_2_ fold difference between active LN and active non-LN SLE patients in the current study. B) Selected urinary analytes that have been previously proposed as LN-specific biomarkers. C) Currently used measures of renal function.
